# Pneumococcal polysaccharide vaccination in rheumatoid arthritis patients receiving tacrolimus

**DOI:** 10.1186/s13075-015-0662-x

**Published:** 2015-06-03

**Authors:** Kiyoshi Migita, Yukihiro Akeda, Manabu Akazawa, Shigeto Tohma, Fuminori Hirano, Haruko Ideguchi, Ryutaro Matsumura, Eiichi Suematsu, Tomoya Miyamura, Shunsuke Mori, Takahiro Fukui, Yasumori Izumi, Nozomi Iwanaga, Hiroshi Tsutani, Kouichirou Saisyo, Takao Yamanaka, Shiro Ohshima, Takao Sugiyama, Yojiro Kawabe, Masao Katayama, Yasuo Suenaga, Akira Okamoto, Hisaji Ohshima, Yasumasa Okada, Kenji Ichikawa, Shigeru Yoshizawa, Kenji Kawakami, Toshihiro Matsui, Hiroshi Furukawa, Kazunori Oishi

**Affiliations:** Japanese National Hospital Organization (NHO, EBM study group), Higashigaoka 2-5-23, Meguro, Tokyo 152-8621 Japan; Clinical Research Center, NHO Nagasaki Medical Center, Kubara 2-1001-1, Omura, 856-8652 Japan; Research Institute for Microbial Diseases, Osaka University, Yamadaoka 3-1, Suita, Osaka 565-8563 Japan; Department of Public Health and Epidemiology, Meiji Pharmaceutical University, Noshio 2-522-1, Kiyose, Tokyo 204-8588 Japan; Infectious Diseases Surveillance Center, National Institute of Infectious Diseases, Toyama 1-23-1, Shinjuku, Tokyo 162-8640 Japan

## Abstract

**Introduction:**

In rheumatoid arthritis (RA) patients receiving immunosuppressive treatments, vaccination against *Streptococcus pneumoniae* is recommended. The objective of the study was to evaluate the effects of tacrolimus (TAC) on immune response following administration of a 23-valent pneumococcal polysaccharide vaccine (PPSV23) in patients with established RA.

**Methods:**

Patients with RA (*n* = 133) were vaccinated with PPSV23. Patients were classified into TAC (*n* = 29), methotrexate (MTX) (*n* = 55), control (*n* = 35), and TAC/MTX (*n* = 14) treatment groups. We measured the concentrations of pneumococcal serotypes 6B and 23F by using an enzyme-linked immunosorbent assay and determined antibody functionality by using a multiplexed opsonophagocytic killing assay, reported as the opsonization index (OI), before and 4 to 6 weeks after vaccination. A positive antibody response was defined as at least a twofold increase in the IgG concentration or as at least a 10-fold increase in the OI.

**Results:**

IgG concentrations and OIs were significantly increased in all treatment groups after PPSV23 vaccination. The TAC treatment group appears to respond in a manner similar to that of the RA control group in terms of 6B and 23F serotype concentration and function. In contrast, the MTX group had the lowest immune response. Patients who received a combination of TAC and MTX (TAC/MTX) also had a diminished immune response compared with those who received TAC alone.

**Conclusions:**

TAC monotherapy does not appear to impair PPSV23 immunogenicity in patients with RA, whereas antibody production and function may be reduced when TAC is used with MTX. Thus, PPSV23 administration during ongoing TAC treatment should be encouraged for infection-prone TAC-treated patients with rheumatic diseases.

**Trial registration:**

University Hospital Medical Information Network Clinical Trials Registry: UMIN000009566. Registered 12 December 2012.

**Electronic supplementary material:**

The online version of this article (doi:10.1186/s13075-015-0662-x) contains supplementary material, which is available to authorized users.

## Introduction

Patients with rheumatoid arthritis (RA) have an increased risk of developing an infectious disease as the underlying conditions and immunomodulatory therapies weaken their immune response [1]. The use of immunosuppressive agents to treat RA may also contribute to this increased risk. Therefore, routine immunizations are important to reduce infection-mediated morbidity and mortality [2, 3]. Given these concerns, the European League Against Rheumatism treatment guidelines have recommended the routine use of pneumococcal vaccines for immune-compromised patients, including those who are receiving tumor necrosis factor (TNF) inhibitors [4, 5]. Notably, the response to vaccination varies among RA patients who are receiving TNF inhibitor therapy with or without concomitant corticosteroids or methotrexate (MTX) or both [6]. A recent meta-analysis demonstrated that patients receiving TNF inhibitors with MTX had a lower response to the pneumococcal vaccine than those receiving TNF inhibitors alone [7]. Furthermore, variable vaccine responses have been reported for patients receiving other biological agents that inhibit T-cell co-stimulation or deplete B cells with or without concomitant corticosteroid or disease-modifying antirheumatic drug (DMARD) treatment or both [8].

We previously reported that, in contrast to other biological treatment agents, tocilizumab, a humanized monoclonal antibody approved for RA treatment, does not impair the immunogenicity of the 23-valent pneumococcal polysaccharide vaccine (PPSV23) in patients with RA [9]. Therefore, we suspect that variation in the immune response to a particular vaccine may depend on both the type of vaccine and which biological agent the patient had received. As intensive research in the field has led to the development of a number of new treatment options for patients with RA, it is essential to investigate the changes in immunogenicity following treatment in order to determine the optimal vaccination options to effectively ward off infection. For example, treatment modalities using calcineurin inhibitors have become available for treatment of RA diseases in the last decade [10]. This includes the T cell-specific immunosuppressant, tacrolimus (TAC). However, studies investigating the production of antibodies following pneumococcal vaccination in patients with RA diseases treated with TAC are limited [11, 12]. Thus, in this study, we have investigated the immunogenicity and tolerability of PPSV23 in Japanese patients with RA following treatment with TAC.

## Methods

### Study design and patient population

We performed a randomized, double-blind, controlled trial. Patients with clinically diagnosed RA were recruited in Japanese National Hospital Organization hospitals across Japan (*n* = 32) from September 2010 to December 2012. Eligible patients were also found to be at risk for developing respiratory infections. Patients with RA were divided into the following groups: (1) patients with rheumatoid lung disease, (2) patients with RA treated with biological agents, and (3) patients who received immunosuppressive agents. Patients were excluded if they had previously received a pneumococcal vaccination. The following parameters were analyzed when the patient was first admitted to the study: swollen joint count, tender joint count, patient global assessment of disease activity, physician global assessment of disease activity, Health Assessment Questionnaire disability index (HAQ-DI) score, serum levels of C-reactive protein (CRP), and Disease Activity Score 28-joint assessment with CRP (DAS28 [CRP]) [13]. This study complies with the principles of the Declaration of Helsinki and was approved by the appropriate institutional review boards at each participating center. All patients provided written informed consent. This study was approved by the ethics committees of the National Hospital Organization central institutional review board (0512014, 2012) and was registered with University Hospital Medical Information Network Clinical Trials Registry (UMIN000009566).

### Intervention

Patients were randomly assigned to receive either 0.5 ml (25 μg) of PPSV23 (Pneumovax NP; Merck Sharp & Dohme Corp., Tokyo, Japan) or 0.5 ml of a placebo (sodium chloride) subcutaneously in the upper arm. The vaccines were prepared in a masked fashion for those who administered it, blinding both the administrator of the vaccine and the patient to the type of vaccine given. Vaccine and placebo were presented in identical single-dose syringes and needle combinations that were labelled with sequential study numbers only. A statistician who was not on the study team carried out the randomization by using a random number table and numbered the containers accordingly.

Patients were instructed to record local reactions (e.g., redness, swelling, and tenderness) and systemic reactions (e.g., fever, nausea, and vomiting). Patients were also monitored for 12 months after enrollment to follow the development of pneumonia, including that stemming from pneumococcal disease. Serum samples were obtained immediately before and 4 to 6 weeks after vaccination and stored at −30 °C until tested. An independent investigator measured the serotype-specific IgG geometric mean concentrations (GMCs) and opsonization indices (OIs) by using the sera samples from patients receiving PPSV23. The measurements were performed in random order, and all clinical data were blinded to prevent biases.

### Enzyme-linked immunosorbent assays for serotype-specific IgG

Enzyme-linked immunosorbent assays (ELISAs) for serotype-specific IgG were performed to measure the concentration of each type of antibody as previously described [9]. Furthermore, to measure IgG specificity for the 6B and 23F serotypes, we specifically performed our ELISAs according to the World Health Organization (WHO) standard procedure that used the international reference serum, 89SF-3 (graciously supplied by Carl E. Frasch). To improve the specificity of the assay, we performed a pneumococcal cell wall polysaccharide (C-PS) and pneumococcal 22F polysaccharide pre-absorption step on the samples. The reference serum was pre-absorbed with only C-PS [14, 15]. Detailed protocols are available at [16].

### Multiplexed opsonophagocytic assays

To measure antibody functionality against pneumococcus, we performed multiplexed opsonophagocytic assays for pneumococcal serotypes 6B and 23F by using differentiated HL-60 cells and antibiotic-resistant target bacteria strains at the Research Institute for Microbial Disease, Osaka University, as previously described [17]. The quality control serum included in each assay was prepared from pooled sera of adults immunized with PPV23. The OI was defined as the serum dilution that led to 50 % death of target bacteria. Opsotiter 3, an Excel-based data-processing program, was used to convert colony counts to OIs in accordance with the WHO protocol available at [18].

### Antibody response

Fold increases relative to pre-vaccination values (ratios of post-vaccination value to pre-vaccination value) were determined. Positive antibody response was defined as at least a twofold increase in IgG concentrations or as at least a 10-fold increase in OIs as described previously [9].

### Statistical analysis

Clinical and demographic data are expressed as mean ± standard deviation or as a percentage. Comparisons were made between the RA treatment groups before and after administration of the vaccine by using an analysis of variance with post-hoc Tukey’s honesty significant difference test. Differences in IgG concentrations or OI before and after vaccination were compared by using the paired-sample *t* test. Multivariate logistic regression analysis with adjustment for baseline characteristics was used to assess the relationship between positive antibody response to both pneumococcal serotype and a set of predictor variables, including age, gender, RA duration, current MTX, prednisolone use, TAC use, and serum IgG levels. A backward stepwise selection procedure was used to select significant independent variables. For all tests, probability values (*P* values) of less than 0.05 were considered statistically significant. All calculations were performed by using Excel Statistical Analysis 2008 (SSRI Co., Ltd., Tokyo, Japan) or PASW Statistics version 20 (SPSS Japan Inc., Tokyo, Japan).

## Results

### Clinical and demographic characteristics

In total, 989 subjects were assessed for eligibility, and 929 patients were recruited and randomly assigned (Additional file 1: Figure S1). Paired serum samples were obtained before and after vaccination from 703 subjects, 353 of which received PPV23. Patients receiving PPSV23 were divided into the following three groups according to their ongoing anti-RA therapy: TAC monotherapy (2.0 ± 0.7 mg/day, TAC group, *n* = 29), MTX monotherapy (7.8 ± 2.4 mg/week, MTX group, *n* = 55), and DMARD (salazosulfapyridine 14/35, bucillamine 5/35, penicillamine 1/35, sodium aurothiomalate 1/35, ignatimod 1/35) or steroid (13/35) treatments (RA control group; *n* = 35). Patients’ clinical and demographic characteristics are shown in Table 1. All participants fulfilled the criteria of safety required for vaccine injection, and no serious side effects were observed after vaccination.

**Table 1:** Clinical and demographic characteristics of rheumatoid arthritis patients prior to pneumococcal vaccination

	RA control	MTX group	TAC group	TAC + MTX group
	*n* = 35	*n* = 55	*n* = 29	*n* = 14
Male/female	12/23	11/44	9/20	1/13
Age in years, mean ± SD	70.54 ± 10.84	63.80 ± 11.50	69.28 ± 9.87	61.00 ± 11.68
Weight in kg, mean ± SD	53.32 ± 9.54	52.87 ± 11.83	52.22 ± 12.24	50.84 ± 9.42
BMI, mean ± SD	21.79 ± 3.47	21.69 ± 3.69	21.84 ± 4.43	21.51 ± 3.23
RA duration in years, mean ± SD	11.66 ± 12.52	14.11 ± 10.90	10.52 ± 9.74	12.14 ± 7.07
MTX dose in mg/week, mean ± SD	-	7.80 ± 2.37	-	8.29 ± 3.22
TAC dose in mg/day, mean ± SD	-	-	2.05 ± 0.74	1.64 ± 0.57
Use of prednisolone, number (%) of patients	21 (60.0)	30 (54.5)	21 (72.4)	6 (42.9)
Prednisolone dose in mg/day, mean ± SD	6.06 ± 4.23	4.98 ± 2.97	3.89 ± 1.99	3.00 ± 1.26
DAS28 (CRP), mean ± SD	2.79 ± 1.17	2.61 ± 0.98	2.79 ± 1.23	2.10 ± 0.71
HAQ-DI, mean ± SD	0.85 ± 0.84	0.62 ± 0.76	0.76 ± 0.88	0.53 ± 0.41
SDAI, mean ± SD	9.03 ± 6.32	8.15 ± 7.33	10.36 ± 10.63	4.60 ± 4.06
CDAI, mean ± SD	7.83 ± 5.40	7.45 ± 6.73	9.68 ± 10.51	3.95 ± 3.84
IP (%)	6 (17.1)	7 (12.7)	13 (44.8)	0
COPD (%)	3 (8.6)	1 (1.8)	2 (6.9)	0
Smoking history (%)	3 (8.6)	5 (9.1)	3 (10.3)	1 (7.1)

Data were obtained immediately before pneumococcal vaccination

*RA* rheumatoid arthritis, *MTX* methotrexate, *TAC* tacrolimus, *SD* standard deviation, *BMI* body mass index, *DAS28* disease activity score 28, *CRP* C-reactive protein, *HAQ-DI* Health Assessment Questionnaire disability index score, *SDAI* simplified disease activity index, *CDAI* clinical disease activity index, *IP* interstitial pneumonia, *COPD* chronic obstructive pulmonary disease

### Serotype-specific IgG concentrations

Four to six weeks after pneumococcal vaccination, the GMCs of both serotype 6B- and 23F-specific IgG were increased in all three groups (*P* < 0.00001, Table 2). However, these elevated GMC responses were not observed in the placebo group (Additional file 2: Table S1). Furthermore, the post-vaccination GMC of serotype 6B-specific antibodies was not significantly different when the three treatment groups were compared, implying that a similar increase was observed regardless of TAC or MTX treatment. However, the concentration of serotype 23F-specific antibodies post-vaccination was significantly higher in patients who received TAC compared with the MTX treatment group (*P* = 0.005).

**Table 2:** Concentrations of pneumococcal polysaccharide antigen serotype-specific IgG antibodies and opsonization indices in the rheumatoid arthritis treatment groups before and after 23-valent pneumococcal polysaccharide vaccination

			MTX group *n* = 55	TAC group *n* = 29	*P* values between treatment groups
IgG GMCs, μg/ml					
6B	Before	0.84 (0.58 to 1.11)	1.42 (0.86 to 1.97)	1.61 (1.11 to 2.11)	NS
	After	4.05 (2.13 to 5.97)*	4.36 (2.17 to 6.55)*	8.35 (2.79 to 13.91)*	NS
	Fold increase	2.38 (1.41 to 5.62)	1.75 (1.15 to 3.11)	2.04 (1.71 to 5.76)	NS
23F	Before	1.17 (0.85 to 1.48)	1.79 (1.33 to 2.25)	1.38 (0.68 to 2.08)	NS
	After	11.61 (4.16 to 19.07)*	7.41 (4.48 to 10.33)*	16.73 (7.81 to 25.65)*	NS
	Fold increase	3.36 (1.85 to 9.42)	2.00 (1.27 to 5.48)	7.63 (3.70 to 18.85)	0.005 (MTX vs. TAC)
GM-OIs					
6B	Before	17.24 (10.96 to 23.53)	150.79 (14.85 to 286.74)	262.89 (56.54 to 469.25)	NS
	After	981.15 (407.24 to 1555.05)*	584.29 (270.29 to 898.28)*	2026.54 (1047.56 to 3005.51)*	0.002 (MTX vs. TAC)
	Fold increase	10.22 (1.92 to 79.48)	2.57 (1.22 to 22.40)	20.80 (3.86 to 70.38)	NS
23F	Before	63.21 (−6.79 to 133.20)	52.11 (14.04 to 90.18)	164.57 (−129.84 to 458.98)	NS
	After	713.49 (307.97 to 1119.01)*	724.56 (336.93 to 1112.19)*	1583.04 (773.21 to 2392.86)*	0.013 (MTX vs. TAC)
	Fold increase	6.86 (2.50 to 27.14)	3.75 (1.47 to 38.32)	64.38 (11.59 to 231.22)	0.048 (MTX vs. TAC)

IgG GMCs and GM-OIs are expressed as the mean (95 % confidence interval). Fold increases are expressed as the median (interquartile range). Differences between pre- and post-vaccination GMCs of serotype-specific IgG were assessed by using a paired-sample *t* test. The three treatment groups were compared by using Kruskal-Wallis test with a Scheffé post-hoc test

*MTX* methotrexate, *TAC* tacrolimus, *GMC* geometric mean concentration, *GM-OI* geometric mean opsonization index, *NS* not significant

*P < 0.00001 compared with pre-vaccination IgG GMCs or GM-OIs

### Opsonophagocytic killing assays

The post-vaccination OIs increased significantly in all treatment groups. The ratios between pre- and post-vaccination are provided in Table 2. Whereas there was no significant difference observed between the TAC and control groups, there were significant differences in the post-vaccination OIs specific to 6B and 23F between the MTX and TAC treatment groups, and the TAC treatment group had higher OIs than the MTX group.

### Antibody response rates

**Fig. 1:**
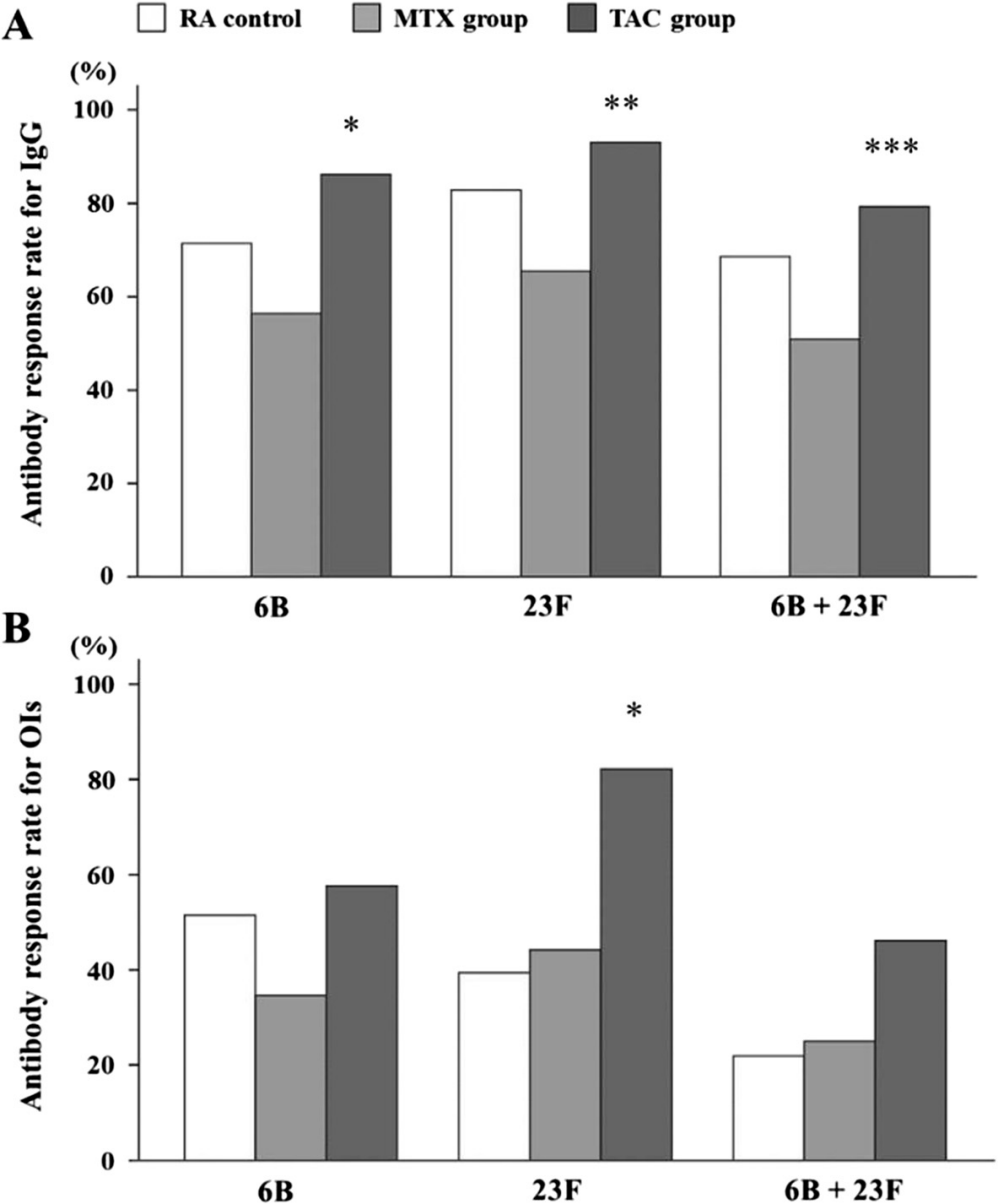
Flow diagram of patient recruitment. Comparison of post-vaccination parameters in patients receiving TAC or MTX monotreatment. **a** Percentage of patients with an increase in 6B and 23F serotype-specific IgG concentration greater than twofold. *P = 0.006, **P = 0.005, ***P = 0.011 for TAC compared with MTX. **b** Percentage of patients with an increase in OI for serotypes 6B and 23F greater than 10-fold. *P = 0.001 (TAC versus RA control) and *P* = 0.001 (TAC versus MTX). Data were compared by using the chi-squared test or Fisher’s exact probability test. *MTX* methotrexate, *OI* opsonization index, *RA* rheumatoid arthritis, *TAC* tacrolimus

The antibody response rates, given as the percentage of patients with a positive antibody response, for patients in the TAC treatment group were comparable to those of the untreated RA control group for both serotype 6B and 23F (Fig. 1a). However, the serotype 6B- and 23F-specific antibody response rates were significantly higher in the TAC group (6B 86.2 %, 23F 93.1 %) compared with the MTX group (6B 56.4 %, 23F 65.5 %; *P* = 0.006 and *P* = 0.005, respectively). Furthermore, the percentage of patients with a positive antibody response for both strains was significantly greater in the TAC treatment group (79.3 %) compared with the MTX group (50.9 %, *P* = 0.011). For OIs specific to serotype 6B and 23F (Fig. 1b), the TAC group (6B 57.7 %, 23F 82.1 %) showed a higher antibody response rate than did the MTX group (6B 34.6 %, *P* = 0.052; 23F 44.2 %, *P* = 0.001). For both strains, a higher proportion of the TAC-treated patients responded to pneumococcal vaccination compared with the patients who received MTX; however, this difference was not significant (46.2 % compared with 25.0 %, respectively; *P* = 0.059).

### Comparison between TAC monotreatment and TAC/MTX combination treatment

**Fig. 2:**
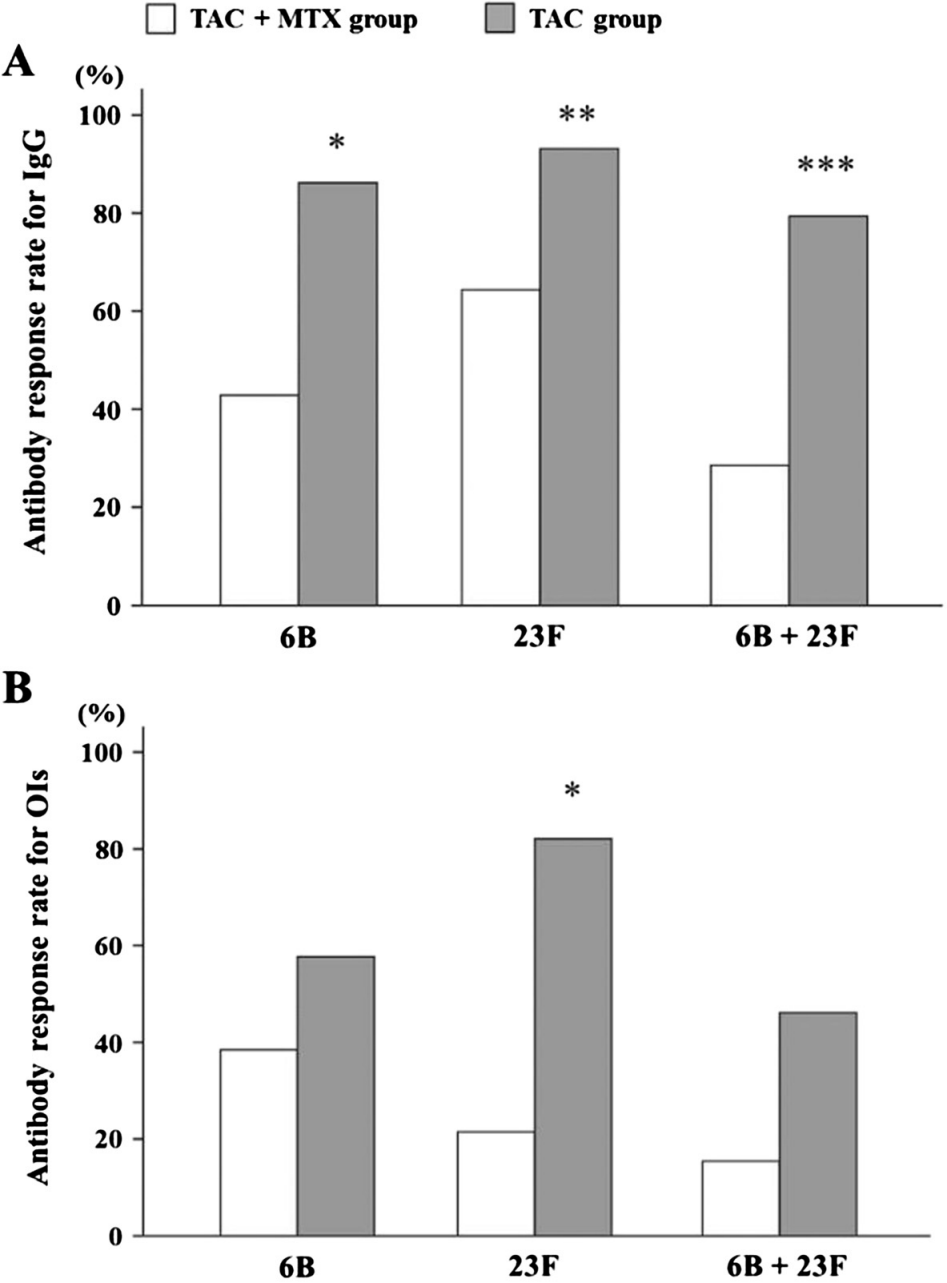
Comparison of post-vaccination parameters in patients receiving TAC monotreatment or TAC/MTX combination treatment. **a** Percentage of patients with an increase in 6B and 23F serotype-specific IgG concentration greater than twofold. *P = 0.005, **P = 0.028, ***P = 0.001, for TAC compared with TAC/MTX. **b** Percentage of patients with an increase in OI for serotypes 6B and 23F greater than 10-fold. *P < 0.0001 for TAC compared with TAC/MTX. Data were compared by using the chi-squared test or Fisher’s exact probability test. *MTX* methotrexate, *OI* opsonization index, *TAC* tacrolimus

Notably, TAC is used primarily as a monotherapy, and the combined use of TAC and MTX is limited in Japan, as highlighted in a recent patient survey from this country [19]. Therefore, we were not surprised that only 14 RA patients receiving both TAC and MTX were enrolled in this study (Table 1). When these patients are compared with those receiving TAC monotherapy, it appears that both groups had a significant increase in the GMC and OI of the 6B and 23F serotypes after PPSV23 vaccination (Table 3). However, the post-vaccination GMC and the OIs for both serotypes were significantly lower in the patients receiving TAC/MTX combination therapy compared with those receiving TAC monotherapy. Similarly, a lower proportion of RA patients receiving TAC/MTX combination therapy had a geometric mean titer increase greater than twofold for both serotypes or an increase in OI greater than 10-fold for the 23F serotype (Fig. 2). These data indicate that the combination of TAC and MTX treatment may cause reduced immune responsiveness against the pneumococcal vaccine PPSV23.

**Table 3:** Concentrations of pneumococcal polysaccharide antigen serotype-specific IgG antibodies and opsonization indices in the rheumatoid arthritis treatment groups before and after 23-valent pneumococcal polysaccharide vaccination

		TAC + MTX group *n* = 14	TAC group *n* = 29	*P* value
IgG GMCs, μg/ml				
6B	Before	2.48 (0.92 to 4.04)	1.61 (1.11 to 2.11)	0.288
	After	11.89 (−2.25 to 26.03)*	8.35 (2.79 to 13.91)***	0.551
	Fold increase	1.39 (1.10 to 2.67)	2.04 (1.70 to 5.40)	0.023
23F	Before	2.02 (0.93 to 3.11)	1.38 (0.68 to 2.08)	0.114
	After	4.94 (2.10 to 7.77)*	16.73 (7.81 to 25.65)***	0.017
	Fold increase	1.85 (1.14 to 3.82)	7.63 (3.70 to 18.85)	*P* < 0.0001
GM-OIs				
6B	Before	167.43 (34.41 to 300.45)	262.89 (56.54 to 469.25)	0.471
	After	715.85 (−138.24 to 1569.94)**	2026.54 (1047.56 to 3005.51)***	0.043
	Fold increase	5.50 (2.31 to 10.60)	20.80 (3.86 to 70.38)	0.053
23F	Before	9.50 (2.60 to 16.40)	164.57 (−129.84 to 458.98)	0.227
	After	91.14 (−13.42 to 195.70)*	1583.04 (773.21 to 2392.86)***	*P* < 0.0001
	Fold increase	3.51 (1.00 to 8.00)	64.38 (11.59 to 231.22)	*P* < 0.0001

IgG GMCs and GM-OIs are expressed as the mean (95 % confidence interval). Fold increases are expressed as the median (interquartile range). Differences between pre- and post-vaccination GMCs of serotype-specific IgG were assessed by using a paired-sample *t* test. *P* values were calculated with chi-squared test for qualitative data

*TAC* tacrolimus, *MTX* methotrexate, *GMC* geometric mean concentration, *GM-OI* geometric mean opsonization index

*P < 0.005, **P < 0.001, ***P < 0.00001, compared with pre-vaccination IgG GMCs or GM-OIs

### Predictive factors for antibody response to PPSV23

In a multivariate logistic regression analysis, TAC use was not identified as the predictive factor for antibody response to pneumococcal vaccination for either IgG concentrations or OIs (Additional file 3: Table S2, Additional file 4: Table S3). The negative association of current MTX use with antibody response was confirmed for both IgG concentrations specific to serotypes 6B and 23F (odds ratio 0.297, 95 % confidence interval 0.129 to 0.684, *P* = 0.004, Additional file 3: Table S2).

## Discussion

Pneumococcal vaccination has been advocated for immunocompromised patients receiving the immunosuppressive RA treatments, such as TAC [20]. TAC is a type of calcineurin inhibitor that blocks T-cell cytokine production and indirectly affects B-cell activation [21]. Thus, a slight decrease in antibody production could be expected from this type of treatment. Notably, TAC has been used previously to prevent organ rejection following transplant and graft-versus-host disease after bone marrow transplants as well as for the treatment of myasthenia gravis, lupus nephritis, and ulcerative colitis [22]. However, the effects of TAC on the immunogenicity of pneumococcal vaccination are inconsistent in transplant recipients receiving treatment [23–25]. For example, Broeders et al. [26] previously assessed the immunogenicity of a polysaccharide vaccine in renal transplant recipients receiving TAC treatment and demonstrated that, although the protective effects of the vaccine were variable, all patients collectively produced suboptimal responses [26]. We suspect that these inconsistencies may be accounted for by the intrinsic effect of B-cell function under these treatment conditions.

In April 2005, TAC was approved as a treatment option for Japanese RA patients who had responded inappropriately to conventional treatments; this approval was subsequently granted in Canada, Korea, and Hong Kong [27]. However, our understanding concerning the interaction and effects of this treatment on vaccine function is limited. Therefore, to evaluate the immune response to PPSV23 in RA patients receiving TAC, we monitored several immune response parameters, including patient health, antibody concentration, and OI, following PPSV23 administration to patients receiving TAC and MTX. To this end, we examined the GMC of serotype 6B and 23F antibodies pre-vaccination and 4 to 6 weeks post-vaccination and found that both serotypes increased significantly in untreated and treated (TAC and MTX monotherapy) patients. It also appears that, for certain serotypes, this PPSV23 vaccine evoked a greater immunogenic response (i.e., a higher concentration and functional response) in RA patients receiving TAC compared with those receiving MTX. These findings suggest that the immunological response against T cell-independent polysaccharide antigens in RA patients following TAC-based immunosuppressive therapy is preserved in comparison with conventional MTX therapy. Combination therapy using both TAC and MTX had a decreased immune response to the PPSV23 vaccine in comparison with TAC alone. Taken together, these data indicate that a greater proportion of TAC-treated patients responded to PPSV23 more efficiently compared with patients who received MTX alone or in conjunction with TAC.

Notably, the pneumococcal vaccine used in this study consists almost exclusively of capsular polysaccharides [28]. This is important to note, as antibody production by B cells often requires “help” from T cells; however, highly repetitive polysaccharide structures have been shown to induce T cell-independent antibody production in B cells [29]. Thus, we suspect that the therapeutic immunosuppression observed in the TAC-treated RA patients in this study affects mainly T-cell function as this would not significantly alter the T cell-independent immune response to this specific pneumococcal vaccination. Furthermore, this conclusion is corroborated by other studies in this field [30]; however, additional work should be conducted by using vaccines with less repetitive polysaccharide structures. Immunogenicity of tetanus toxoid and 23-valent pneumococcal vaccinations was impaired in healthy subjects receiving abatacept, a selective T-cell co-stimulation modulator [31]. Also, treatment with abatacept was associated with diminished antibody response of pneumococcal conjugate vaccine (PCV 7) [8]. Our data were not consistent with this previous study by Tay et al., who used the same PPSV23 vaccination [31]. The differences in the subjects (healthy subjects versus RA patients) or T cell-targeted treatments (abatacept versus tacrolimus) may contribute to the discrepancies between our data and those of a previous study [31]. Protein-conjugated vaccines, which induce T cell-dependent immune response, may induce superior immune responses compared with the polysaccharide vaccine. However, further investigations for the immunogenicity of these T cell-dependent vaccines under T cell-targeted immunosuppressive treatments will be needed.

The primary limitation of this study is the relatively small number of patients with RA in each group, particularly for TAC/MTX combination treatment (*n* = 14). Sample size calculations were based on our previous study obtained RA patients receiving PPSV23 vaccination [9]. In this study, RA patients receiving MTX achieved the fold induction of 6B-specific (mean ± SD; 1.6 ± 0.8) and 23F-specific (mean ± SD; 2.1 ± 1.0) IgG after 4 to 6 weeks from vaccination. Under this assumption, 33 (for 6B) or 27 (for 23F) evaluable patients samples for each group were needed with a one-tailed *t* test with significance of 0.05 and 80 % power to detect 40 % reduction of GMC fold induction between the MTX group and the TAC/MTX group. Furthermore, we choose to investigate serotypes 6B and 23F because they are the main causative serotypes of penicillin-resistant pneumococcal pneumonia in Japan [32]. Although this allowed us to focus solely on these important serotypes, the effects of TAC on other serotypes during the PPSV23 vaccine-induce immune response are still unknown. Lastly, the antibody concentrations necessary for protection against invasive pneumococcal disease in adults have not been clearly defined [33]. Although we used the thresholds provided by previous studies, namely a twofold increase in IgG concentration and a 10-fold increase in the OI, to measure the positive antibody response to PPSV23 in this study, the suitability of these thresholds to predict prevention of pneumococcal infection has not been widely investigated.

## Conclusions

We have shown that pneumococcal vaccination for RA patients receiving TAC treatment induced an adequate immunogenic response to the PPSV23 vaccine. However, diminished responsiveness to PPSV23 was observed in patients receiving TAC and concomitant MTX. On the basis of these data, we believe that pneumococcal vaccination and TAC treatment should be used more frequently in infection-prone patients with RA.

## Additional files

Additional file 1: Figure S1. Flow diagram of patient recruitment. Additional file 2: Table S1. Concentrations of pneumococcal polysaccharide antigen serotype-specific IgG antibodies and opsonization indices in the rheumatoid arthritis (*RA*) patients before and after placebo injection. Additional file 3: Table S2. Predictors of positive IgG response for both 6B and 23F among patients with rheumatoid arthritis (*RA*). Additional file 4: Table S3. Predictors of positive opsonization index (*OI*) response for both 6B and 23F among patients with rheumatoid arthritis (*RA*).

## Abbreviations

C-PS: Cell wall polysaccharide
CRP: C-reactive protein
DMARD: Disease-modifying antirheumatic drug
ELISA: Enzyme-linked immunosorbent assay
GMC: Geometric mean concentration
MTX: Methotrexate
OI: Opsonization index
PPSV23: 23-valent pneumococcal polysaccharide vaccine
RA: Rheumatoid arthritis
TAC: Tacrolimus
TNF: Tumor necrosis factor
WHO: World Health Organization
