# Supplementary figures and images for: Pneumococcal polysaccharide vaccination in rheumatoid arthritis patients receiving tacrolimus
Source: Arthritis Res Ther. 2015 Jun 3;17(1):149. doi: 10.1186/s13075-015-0662-x (PMC4481124; doi:10.1186/s13075-015-0662-x)

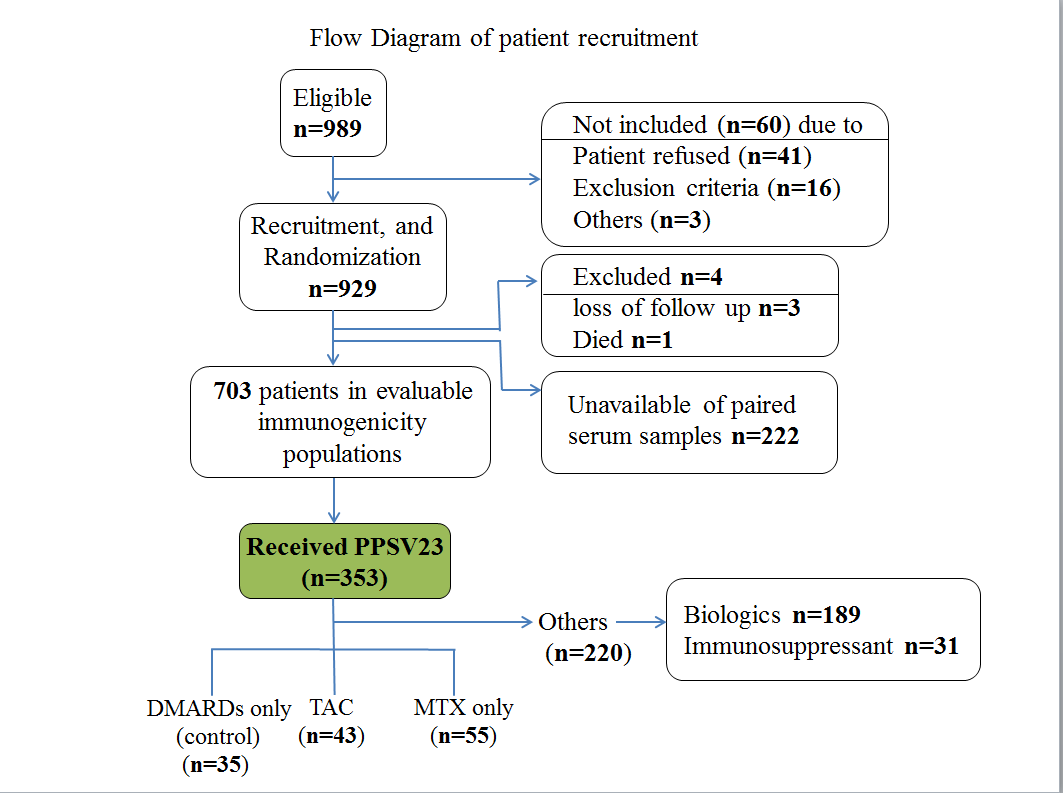

Supplement: Additional file 1: Figure S1. — Flow diagram of patient recruitment. [file 13075_2015_662_MOESM1_ESM.tiff]

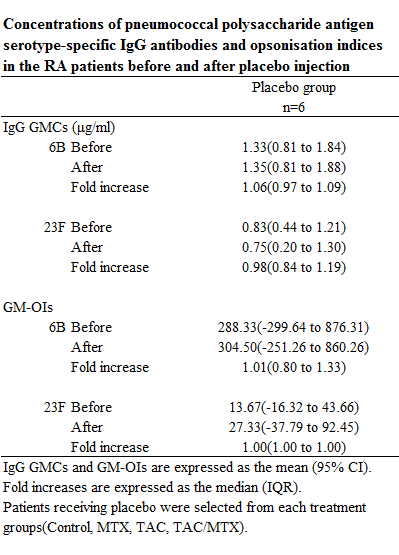

Supplement: Additional file 2: Table S1. — Concentrations of pneumococcal polysaccharide antigen serotype-specific IgG antibodies and opsonization indices in the rheumatoid arthritis (RA) patients before and after placebo injection. [file 13075_2015_662_MOESM2_ESM.tiff]

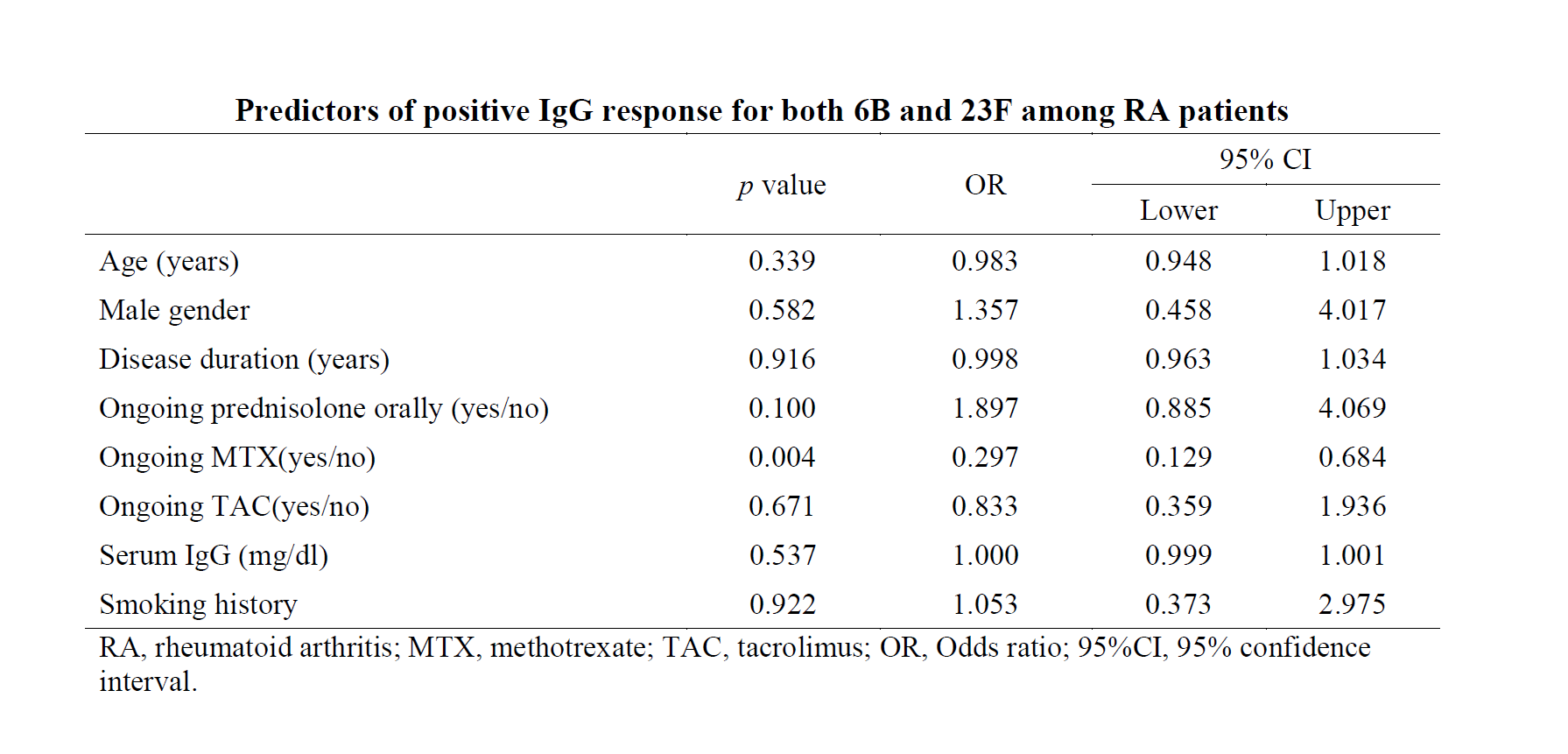

Supplement: Additional file 3: Table S2. — Predictors of positive IgG response for both 6B and 23F among patients with rheumatoid arthritis (RA). [file 13075_2015_662_MOESM3_ESM.tiff]

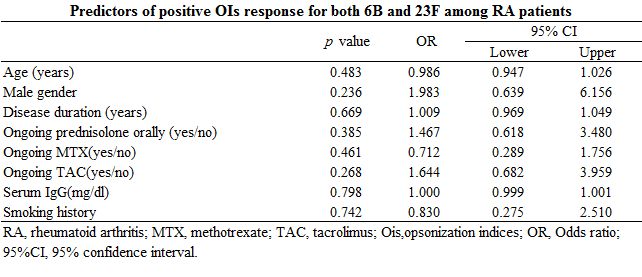

Supplement: Additional file 4: Table S3. — Predictors of positive opsonization index (OI) response for both 6B and 23F among patients with rheumatoid arthritis (RA). [file 13075_2015_662_MOESM4_ESM.tiff]
